# Editorial: Natural compounds as scaffolds for the discovery of new anti-cancer drugs: Focus on terpenoids and flavonoids

**DOI:** 10.3389/fphar.2022.984849

**Published:** 2022-08-11

**Authors:** Valeria P. Sülsen, Constantinos M. Athanassopoulos, José M. Padrón, Rodrigo E. Tamura

**Affiliations:** ^1^ Consejo Nacional de Investigaciones Científicas y Técnicas (CONICET)—Universidad de Buenos Aires, Instituto de Química y Metabolismo del Fármaco (IQUIMEFA), Junín, Buenos Aires, Argentina; ^2^ Universidad de Buenos Aires, Facultad de Farmacia y Bioquímica, Cátedra de Farmacognosia, Junín, Buenos Aires, Argentina; ^3^ Department of Chemistry, University of Patras, Patras, Greece; ^4^ BioLab, Instituto Universitario de Bio-Orgánica Antonio González (IUBO-AG), Universidad de La Laguna, La Laguna, Spain; ^5^ Departamento de Ciências Biológicas, Universidade Federal de São Paulo, Diadema, SP, Brazil

**Keywords:** sesquiterpene lactones, diterpenoids, flavonoids, tumor, neoplasm, anti-cancer, natural compounds

Cancer is a leading cause of death worldwide and it is estimated that there were nearly 10 million deaths in 2020 due to cancer (WHO, 2022). This disease is characterized by an abnormal proliferation of tumor cells in the body, resistance to cell death and other hallmarks that lead to the development of a tumor mass with potential to metastasize and spread to the body. Although chemotherapy is one of the best treatments for cancer, the drugs currently in use have serious side effects and have shown increasing resistance. For this reason, it is necessary to look for new, more effective and selective drugs with fewer adverse effects.

Natural products have played an important role in the discovery and development of new drugs. Many of the drugs that are currently used in therapeutics originate from natural compounds. Despite advances and the development of synthetic pharmaceutical chemistry, drugs derived from natural sources continue to hold a prominent position. The great chemical diversity they present determines that there is a greater possibility of finding new molecules with unique structures and potential biological activities (Davison and Brimble, 2019).

More than 60% of the agents available to treat cancer are connected to natural sources (Newman and Cragg, 2020). Examples include vincristine, vinblastine, podophyllotoxin, paclitaxel, among others.

Natural compounds such as terpenoids and flavonoids have attracted the interest of many researchers. Sesquiterpene lactones and diterpenoids have shown to be promising groups for the development of potential antitumor agents due to their preferential selectivity over certain tumors and cell lines additionally acting on specific signaling pathways (Laurella et al., 2022; Deng et al., 2022; Guo et al., 2022). Flavonoids have demonstrated a preventive effect in the development and progress of cancer and metastasis (Guo et al., 2022; Ferreira et al., 2022). These compounds have also been shown to improve the effectiveness of current chemotherapies.

Our Research Topic collected studies on natural compounds as scaffolds for the discovery of new anticancer drugs, focusing on terpenoids and flavonoids, phytochemical groups of great interest. Natural compounds as well as semisynthetic derivatives with activity on any type of cancer has been considered.

In this sense, Orozco Barocio et al. evaluated the effect of *Lophocereus schottii* extract and fractions on murine lymphoma cells. *L. schottii* is a cactus used in the Mexican traditional medicine as anticancer. The authors demonstrated that the extract and fractions have antiproliferative activity against L5178Y cells. The polar fraction showed the best effect (IC_50_ = 15 μg/ml), being three times more active than cyclophosphamide. The analysis of the fraction by ultra-performance liquid chromatography-mass spectrometry (UPLC-MS) revealed the presence of flavonoids, alkaloids, terpenoids and sterols.

On the other hand, Yang et al., demonstrated that *Platycodon grandiflorum* immunomodulates T cells, reduces the expression of the inhibitory receptor programmed death-1 (PD-1) on the surface of CD8^+^ T cells and shows cytotoxic activity on non-small cell lung cancer (NSCLC). *In vitro* studies showed that *P. grandiflorum* inhibited the growth of LLC, H1975, A549, CT26, and B16-F10 cell lines. In the *in vivo* assay *P. grandiflorum* slowed down the process of tumor development, reducing tumor growth and increasing the survival. The antitumor effect of the combination of Platycodin D and Platycodin D3 has also been confirmed.

Paeoniflorin is a terpene glycoside that has anticancer properties. Wang et al. studied the mechanisms of paeoniflorin in epithelial-to-mesenchymal transition (EMT) and angiogenesis in glioblastoma cells. The authors demonstrated that this compound inhibited EMT *via* downregulating c-Met signaling. Paeoniflorin also showed anti-angiogenic effects by suppressing cell proliferation, migration, invasion and tube formation. Additionally, this compound induced autophagy activation involving mTOR/P70S6K/S6 signaling and promoted c-Met autophagic degradation. Paeoniflorin also suppressed mesenchymal makers and inhibited angiogenesis on an *in vivo* model.

Finally, Dey et al. reviewed the anticancer potential of the diterpenoid quinone salvicine. This compound has been demonstrated to be active both *in vitro* and *in vivo* and also on multidrug-resistant (MDR) cells. Salvicine decreases lung metastatic formation in the MDA-MB-435 lung cancer cell line. Several studies have shown that salvicine-induced ROS play an important role in the anticancer-mediated signaling pathway. This compound would act as a modulator of Topoisomerase II and ROS signaling cascade. The authors highlight the potential of salvicine as an alternative option for cancer treatment.

In conclusion, this Research Topic has provided some new studies and reviews in the field of anticancer natural products. Activity of extracts and natural compounds as well as the description of their mechanisms of action have been described. Flavonoids and terpenoids are natural compounds that have been considered of great interest for anticancer drug development. These molecules can target different cancer hallmarks, inhibiting proliferation, angiogenesis and invasion, modulating cancer cell cycle, promoting programmed cell death and having immunomodulatory activities directed against different tumor cells. Further exploration, supported by broad interdisciplinary studies (medicinal chemistry, pharmacology, molecular, and cellular biology) should be conducted with these two phytochemical groups in order to contribute to find new therapeutic options to fight against cancer.
